# Characterization of Mutations in DNA Gyrase and Topoisomerase IV in Field Strains and In Vitro Selected Quinolone-Resistant *Mycoplasma hyorhinis* Mutants

**DOI:** 10.3390/antibiotics11040494

**Published:** 2022-04-07

**Authors:** Jun Li, Yanna Wei, Jia Wang, Yao Li, Guoqing Shao, Zhixin Feng, Qiyan Xiong

**Affiliations:** 1 Jiangsu Key Laboratory for Food Quality and Safety-State Key Laboratory Cultivation Base of Ministry of Science and Technology, Institute of Food Safety and Nutrition, Jiangsu Academy of Agricultural Sciences, Nanjing 210040, China; lijunjaas@126.com (J.L.); gqshaonj@163.com (G.S.); fzxjaas@163.com (Z.F.); 2 Institute of Veterinary Medicine, Jiangsu Academy of Agricultural Sciences, Nanjing 210014, China; weiyanna05@163.com (Y.W.); dirkwang@126.com (J.W.); liyao960410@126.com (Y.L.); 3 College of Veterinary Medicine, Nanjing Agricultural University, Nanjing 210014, China

**Keywords:** in vitro, selection, fluoroquinolones, antimicrobial resistance, QRDRs

## Abstract

*Mycoplasma hyorhinis* is ubiquitous in swine, and it is a common pathogen of swine that causes polyserositis, arthritis, and maybe pneumonia. Fluoroquinolones are effective antimicrobials used for the treatment of mycoplasmal infection. However, a decrease in fluoroquinolones susceptibility in mycoplasma was observed. The molecular mechanisms have been studied in many mycoplasma species, while the mechanism in *M. hyorhinis* is still unknown. This study aimed to illustrate the in vitro development of fluoroquinolone resistance in *M. hyorhinis* and unveil the resistance mechanisms in both in vitro selected mutants and field strains. Seven ciprofloxacin-sensitive *M. hyorhinis* isolates were chosen to induce the fluoroquinolone resistance in vitro, and the point mutations in the quinolone resistance-determining regions (QRDRs) were characterized. The substitutions first occurred in ParC, resulting in a 2- to 8-fold increase in resistance, followed by additional mutations in GyrA and/or ParE to achieve a 32-fold increase. The mutations occurred in hot spots of QRDRs, and they were diverse and variable, including five in ParC (Ser80Phe, Ser80Tyr, Phe80Tyr, Glu84Gly, and Glu84Lys), four in GyrA (Ala83Val, Ser84Pro, Asp87Tyr, and Asp87Asn) and one in ParE (Glu470Lys). Target mutations in field strains were observed in the ParC (Ser80Phe, Ser81Pro, and Glu84Gln) of isolates with MIC_CIP_ = 2 μg/mL. This study characterized the point mutations in the QRDRs of *M. hyorhinis* and could be useful for the rapid detection of fluoroquinolone resistance in *M. hyorhinis* field isolates.

## 1. Introduction

*Mycoplasma hyorhinis* (*M. hyorhinis*) is a small, pleomorphic, and cell wall-less bacterium which was first isolated in 1953 [1,2]. *M. hyorhinis* is ubiquitous in swine and commonly inhabits the ciliated upper respiratory tract [3,4]. Most colonized pigs show no visible clinical symptoms. Under certain conditions, *M. hyorhinis* can cause systemic infections, including polyserositis, arthritis, abortion, otitis, conjunctivitis, and maybe pneumonia [5,6]. Additionally, *M. hyorhinis* has also been reported to be linked with human cancer, such as gastric, esophageal, lung, breast, glioma, and colon cancers [7]. In addition, *M. hyorhinis* is one of the most common mycoplasmal contaminants in cultured cell lines [8].

Fluoroquinolones (enrofloxacin, ciprofloxacin, and marbofloxacin), macrolides (tylosin, tilmicosin, and tiamulin), and tetracyclines (oxytetracycline and doxycycline) are effective antimicrobials against *Mycoplasma* spp. [9]. These antimicrobials are frequently applied in animal husbandry for treating bacterial infections as well as mycoplasmal infections. Additionally, they are used to prevent and eliminate the contamination of mycoplasma in the cell cultures [10]. However, several studies have reported a decrease in fluoroquinolones susceptibility of mycoplasma species when comparing old with recent strains [9,11]. Fluoroquinolones inhibit bacterial/mycoplasmal replication by acting at DNA gyrase (coding by *gyrA* and *gyrB*) and topoisomerase IV (coding by *parC* and *parE*) [12]. Mutations in these genes have been proven to be related to the acquirement of quinolone resistance in *Mycoplasma* spp. [9].

The quinolone resistance mechanisms have been illustrated in many *Mycoplasma* spp., such as *M. agalactiae* [13], *M. gallisepticum* [14], *M. synoviae* [15], *M. hyopneumoniae* [16] and *M. bovis* [17]. However, the quinolone resistance mechanism in *M. hyorhinis* was still unknown. Hence, this study aimed to illustrate the in vitro developing process of fluoroquinolone resistance in *M. hyorhinis* and unveil the molecular mechanisms by using both in vitro selected mutants and field strains, especially the occurrence and contributions of specific mutations in the QRDRs.

## 2. Results

### 2.1. Antimicrobial Susceptibility of M. hyorhinis Field Strains

In the present study, the minimal inhibitory concentration (MIC) values of reference strain BTS-7 were similar with previous studies [9,11,18], with MICs of ciprofloxacin at 0.25 μg/mL, doxycycline at 0.125 μg/mL, florfenicol at 1 μg/mL, lincomycin at 0.25 μg/mL, oxytetracycline at 0.25 μg/mL, tiamulin at 0.015 μg/mL, tilmicosin at 0.5 μg/mL, tylosin at 0.03 μg/mL and tylvalosin at 0.015 μg/mL, indicating good reproducibility of the test in our laboratory conditions.

**Figure 1:**
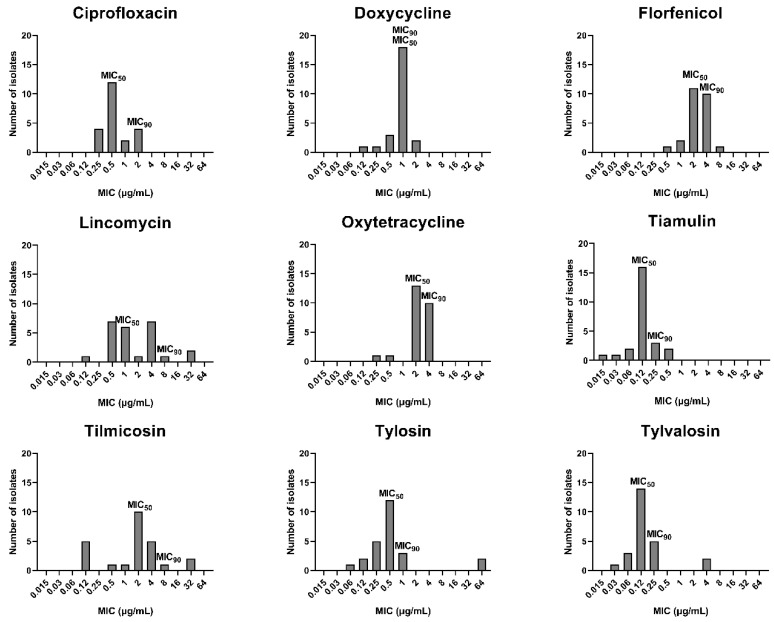
The MIC distributions of 25 strains of *M. hyorhinis* field isolates. MIC_50_ for the lowest concentrations that inhibit 50% of bacterial isolates. MIC_90_ for the lowest concentrations that inhibit 90% of bacterial isolates.

MIC distributions of the 25 field strains of *M. hyorhinis* to 9 antimicrobial agents are shown in Figure 1. All the isolates had low MIC values to ciprofloxacin (≤2 μg/mL), doxycycline (≤2 μg/mL), oxytetracycline (≤4 μg/mL), tiamulin (≤0.5 μg/mL), and tylvalosin (≤4 μg/mL). Of these antimicrobials, tiamulin and tylvalosin showed the best antimicrobial activity, with the lowest MIC_50_ at 0.12 μg/mL and MIC_90_ at 0.25 μg/mL. In the cases of lincomycin, tilmicosin, and tylosin, there were several strains showing high MIC values (≥32 μg/mL), which might be resistant isolates. For tylvalosin, the MIC distribution divided into two populations (<0.5 µg/mL and >2 µg/mL). The field isolates with MIC_TVN_ = 4 μg/mL were less susceptible to tylvalosin, which might be a sign of resistance. In total, the MIC distributions of ciprofloxacin, doxycycline, florfenicol, oxytetracycline, and tiamulin were clustered, while the MIC distributions of lincomycin and tilmicosin were relatively scattered.

### 2.2. In Vitro Selection of Fluoroquinolone-Resistant Mutants of M. hyorhinis

Seven strains of ciprofloxacin-sensitive *M. hyorhinis* (Mhr-JS-2016-47, Mhr-JS-2010-36, Mhr-JS-2010-37, Mhr-JS-2011-40, Mhr-JS-2018-53, Mhr-JS-2014-43, Mhr-JS-2015-44; MIC_CIP_ = 0.5 μg/mL), which presented different antimicrobial-susceptible phenotypes, were chosen to induce the resistance to fluoroquinolone in this study (Table 1). Although 5 passages were performed for all the parental strains, the results of selection showed marked differences between the strains. Finally, the highest ciprofloxacin MIC for the mutants derived from Mhr-JS-2016-47, Mhr-JS-2014-43, Mhr-JS-2015-44, Mhr-JS-2010-37, and Mhr-JS-2011-40 was 16 μg/mL, compared with 8 μg/mL for the mutants derived from Mhr-JS-2010-36 and Mhr-JS-2018-53. In summary, fluoroquinolone-resistant mutants were successfully selected from all 7 isolates of ciprofloxacin-sensitive *M. hyorhinis*, and the MIC increased 16–32 folds in comparison with the corresponding parental strains.

### 2.3. Characterization of Mutations in QRDRs in Both In Vitro Selected Mutants and Field Strains

The MIC results and DNA changes related to in vitro selected fluoroquinolone resistance are shown in Table 2. The mutations in QRDRs occurred in the sequence of *parC*, *parE,* and *gyrA* (except *gyrB*). When the MIC of the mutants increased by 2-fold, the mutations first occurred at the amino acid position 80 (Ser80Phe, Ser80Tyr, and Phe80Tyr) or 84 of ParC (Glu84Gly and Glu84Lys). Both mutations of Ser80Tyr (Mhr-JS-2014-43 and Mhr-JS-2018-53) and Glu84Lys (Mhr-JS-2010-37 and Mhr-JS-2011-40) occurred in two isolates, while other mutations were just found in one strain. The mutation in ParE was rare; it just appeared at the point of amino acid position 470 (Glu470Lys) and only occurred in two isolates (Mhr-JS-2016-47 and Mhr-JS-2018-53).

Multiple mutations in different QRDRs were needed for the fluoroquinolone-resistant mutants to achieve higher resistant levels. The mutations in *gyrA* were found in the resistant mutants with higher MIC values (≥2 μg/mL). The mutations in GyrA mainly occurred in 3 hot points, including amino acid positions of 83 (Ala83Val), 84 (Ser84Pro), and 87 (Asp87Tyr and Asp87Asn). Ala83Val was the most common mutation point, which was found in three isolates (Mhr-JS-2010-36, Mhr-JS-2015-44, and Mhr-JS-2011-40), followed by Asp87Asn, which was found in the isolates of Mhr-JS-2014-43 and Mhr-JS-2010-37.

The target mutations in QRDRs were also determined for all the field isolates of *M. hyorhinis.* The genome sequence (NZ_KB911485.1) of ciprofloxacin-sensitive BTS-7 was used as a reference to compare with the field isolates. The results showed that point mutations were only observed in the ParC of *M. hyorhinis* isolates, with the highest MIC_CIP_ at 2 μg/mL. Specifically, Ser80Tyr was detected in Mhr-JS-2016-46, Ser81Pro was observed in Mhr-JS-2011-39, and Glu84Gln was found in Mhr-JS-2010-35 and Mhr-JS-2018-53.

## 3. Discussion

The infection of *M. hyorhinis* is ubiquitous in swine; however, no effective vaccine is commercially available against *M. hyorhinis* infection at present. Antimicrobial is the main intervention used to minimize the infection. However, because the antimicrobial susceptibility testing of mycoplasmas is difficult and time-consuming, the relevant MIC data concerning *M. hyorhinis* are scarce. In the present study, we found that the field *M. hyorhinis* isolates were most sensitive to tiamulin and tylvalosin. Ciprofloxacin, doxycycline, florfenicol, and oxytetracycline showed moderate antimicrobial activities. There were several isolates showing resistance to lincomycin, tilmicosin, and tylosin, with MICs above 32 μg/mL.

The in vitro antimicrobial susceptibility data from this study was consistent with previous studies. The MIC values of various antimicrobials against *Mycoplasma hyorhinis* in these years have been comprehensively reviewed by Gautier-Bouchardon, A.V. [9]. In 2020, Ruben S. Rosales et al. reported that 48 strains of *M. hyorhinis* collected from southern Europe showed the highest sensitivity to tylvalosin (MIC_50_ = 0.016 μg/mL; MIC_90_ = 0.125 μg/mL), valnemulin (MIC_50_ = 0.016 μg/mL; MIC_90_ = 0.03 μg/mL), and tiamulin (MIC_50_ = 0.125 μg/mL; MIC_90_ = 0.5 μg/mL) [19]. There were also isolates showing decreased susceptibility to lincomycin with MICs > 64 μg/mL. Another recent study from central Europe reported the antibiotic susceptibility profiles of 38 Hungarian *M. hyorhinis* strains isolated between 2014 and 2017 [11]. Low MIC values for tetracyclines (MIC_50_ 0.078 μg/mL for doxycycline and ≤0.25 μg/mL for oxytetracycline) and pleuromutilins (MIC_50_ 0.156 μg/mL for tiamulin and ≤0.039 μg/mL for valnemulin) were observed. There were also numerous isolates showing decreased susceptibility to macrolides and lincomycin (MIC_90_ > 64 μg/mL for tylosin, tilmicosin, tulathromycin, gamithromycin, and lincomycin, 8 μg/mL for tylvalosin). Twelve field isolates of *M. hyorhinis* from Korea were most sensitive to tylvalosin (MIC_50_ 0.06 μg/mL and MIC_90_ 0.12 μg/mL) and tiamulin (MIC_50_ 0.12 μg/mL and MIC_90_ 0.25 μg/mL). Some strains exhibited higher MIC value to chlortetracycline (MIC > 64 μg/mL) [20]. For the antimicrobial susceptibility data of *M. hyorhinis* before 2000, tetracyclines and macrolides were the most effective antibiotic classes [21].

Based on our antimicrobial susceptibility results, seven strains of ciprofloxacin-sensitive *M. hyorhinis*, which presented different antimicrobial-susceptible phenotypes, were chosen to induce the resistance to fluoroquinolone and unveil its mechanisms. The molecular mechanisms of fluoroquinolones resistance of *Mycoplasma* spp. have been reported in *M. agalactiae* [13], *M. gallisepticum* [14], *M. synoviae* [15], *M. hyopneumoniae* [16], *M. bovis* [17], which mainly involve the target mutations in the QRDRs. However, the molecular mechanisms of *M. hyorhinis* resistance against fluoroquinolones have not been reported before. In this study, we were able to select quinolone-resistant *M. hyorhinis* mutants after serial passages in stepwise increased concentrations of ciprofloxacin. We first characterized the point mutations in the QRDRs of *M. hyorhinis* mutants with elevated MIC values to fluoroquinolones, and the results showed that the molecular mechanisms involved separate sequential mutations in topoisomerase IV and DNA gyrase. The substitutions included 5 in ParC [3 in codon 80 (Ser80Phe, Ser80Tyr, and Phe80Tyr), 2 in codon 84 (Glu84Gly and Glu84Lys)], 4 in GyrA [2 in codon 87 (Asp87Tyr and Asp87Asn), 1 in codon 83 (Ala83Val), 1 in codon 84 (Ser84Pro)], and 1 in ParE (Glu470Lys).

In this study, the primacy of ParC mutations over those in GyrA strongly suggested that topoisomerase IV might be the primary target of fluoroquinolones in *M. hyorhinis*, which was consistent with the reports of other *Mycoplasma* species, such as *M. agalactiae* [13], *M. hominis* [22], and *M. bovis* [23]. When selected with ciprofloxacin, the amino acid substitution at position 80 or 84 of ParC was the first change that appeared, leading to the increment of MIC values at 2-4 folds. However, when the amino acid position 84 of Glu was replaced with Gly for the isolate of Mhr-JS-2010-36, the MIC increased to 8 μg/mL (8-fold). The substitutions of Ser80Phe and Ser80Tyr have been previously described in fluoroquinolone-resistant isolates of *Mycoplasma hyopneumoniae* [24], while the point mutations at amino-acid position 84 (Glu84Gly and Glu84Lys) were reported in *Mycoplasma gallisepticum* [25].

In order to survive under a higher concentration of ciprofloxacin, the QRDRs in the *M. hyorhinis* underwent second-, even third-step mutations, mainly in the GyrA subunit and less in the ParE. The point mutation of Glu470Lys in ParE only occurred in 2 lineages (Mhr-JS-2016-47 and Mhr-JS-2018-53), leading to the increase of MIC_CIP_ by 4-fold. This novel mutation is described for the first time for *Mycoplasma* spp. in the present study. All the in vitro selected quinolone-resistant *M. hyorhinis* mutants had both mutations in the ParC and GyrA subunits. Double/triple mutations increased the ciprofloxacin MICs of in vitro selected mutants by 16-32 fold compared to the parental strain, which indicated the cumulative effects of the mutations on the MICs. In addition, the *gyrA*-mediated resistance was only detectable in the *parC* mutants. The GyrA mutations were found at positions of hot spots, such as amino acid positions of 83 (Ala83Val), 84 (Ser84Pro), and 87 (Asp87Tyr and Asp87Asn), leading to a 2- to 16-fold increase in the MICs. Of all the substitutions, only the Ala83Val mutation was previously reported by another swine mycoplasmas of *M. hyopneumoniae* [16].

The comparison of several independent lineages indicates that these mutations accumulate following a similar trajectory: first in ParC, resulting in a 2- to 8-fold increase in resistance, followed by additional mutations in GyrA and/or ParE to reach up to a 32-fold increase. The mutations occurred in hot spots of QRDRs; moreover, the mutated amino acids were variable and diverse. To validate the contributions of in vitro selected target mutations in the fluoroquinolone resistance of *M. hyorhinis*, point mutations in the QRDRs of field isolates were also determined. The point mutations were only observed in the ParC subunit (Ser80Tyr, Ser81Pro, and Glu84Gln) of field isolates with the highest MIC_CIP_ (2 μg/mL), which may be due to the relative low MIC level (2 μg/mL) of field strains compared to the in vitro selected mutants (16 μg/mL). The amino-acid substitution of Ser80Tyr was found in both the laboratory-derived resistant mutants of Mhr-JS-2018-53 and Mhr-JS-2014-43 and the field isolate of Mhr-JS-2015-45. Other mutations such as Ser81Pro were previously reported in *M. gallisepticum* [25] and *M. synoviae* [15], while Glu84Gln has been described in *M. gallisepticum* [25]. The in vitro study of mutations of QRDRs could be useful for the establishment of methods for rapid detection of fluoroquinolone resistance in *M. hyorhinis* field isolates.

## 4. Materials and Methods

### 4.1. Mycoplasma Isolates and Growth Conditions

A total of 25 *M. hyorhinis* field isolates were included in this study. They originated from different pig farms in the Jiangsu province of China from July 2010 to October 2018. The *M. hyorhinis* isolates were confirmed by nested PCR assay of the p37 gene and cultured at 37 °C in KM2 broth medium or on KM2 agar [26]. The growth of *M. hyorhinis* was evaluated by the number of color-changing units (CCU) (red to yellow shift), which was calculated by the microplate dilution method [27]. Type stain of BTS-7 (ATCC 17981, NZ_KB911485.1) was used as control. For determination of minimal inhibitory concentration (MIC) values and in vitro selection of fluoroquinolone-resistant mutants, thallium acetate and penicillin were excluded from the KM2 broth culture medium.

### 4.2. Antimicrobial Susceptibility Testing

The antimicrobial susceptibility of *M. hyorhinis* to ciprofloxacin, tylosin, tiamulin, doxycycline, tylvalosin, lincomycin, oxytetracycline, tilmicosin, and florfenicol were determined using the broth microdilution method, according to the recommendation of Hannan [27]. All the tested antimicrobials were purchased from Solarbio Science & Technology Co., Ltd. (Beijing, China), and were of ≥98% purity. For the antimicrobial susceptibility testing, 2-fold dilutions of each antimicrobial at the range of 0.015 μg/mL to 128 μg/mL were freshly prepared in KM2 broth medium. Then the antimicrobials were mixed with an equal volume (100 μL) of *M. hyorhinis* cultures at 10^6^ CCU/mL in a sterilized 96-well microtiter plate. The final concentrations of tested antimicrobials ranged from 0.0075 μg/mL to 64 μg/mL. The final MIC value for each test was defined as the lowest concentration of antimicrobials with no visible growth in the KM2 broth (no red to yellow color change). The reference strain of BTS-7 was used as the quality control in the MIC determination. Each MIC testing was performed with three independent repeats.

### 4.3. Selection of Fluoroquinolone-Resistant Mutants

A total of 7 strains of ciprofloxacin-sensitive *M. hyorhinis* were chosen to induce resistance to fluoroquinolone in this study (Table 1). The in vitro selection of fluoroquinolone-resistant mutants process was conducted according to previously described methods with minor modifications [13,14]. *M. hyorhinis* from a 1 mL of mid-exponential culture (10^6^ CCU) were centrifuged for 15 min at 8000 g at room temperature and resuspended in 1 mL of KM2 medium containing ciprofloxacin at the concentration of 1/2 MIC. After the medium’s color changed from red to yellow, the culture was centrifuged for 15 min at 8000 g at room temperature. Then, the sediment was resuspended in 100 μL of KM2 broth medium and plated onto KM2 agar plates supplemented with an equivalent concentration of ciprofloxacin (1/2 MIC). Then, a single colony was picked and propagated in 2 mL of KM2 broth medium. After the culture medium showed a red to yellow shift, one aliquot of the *M. hyorhinis* was preserved and stored at −80 °C for further analysis, while the other aliquot was tested for its CCU and adjusted to 10^6^ CCU, then centrifuged and resuspended in 1 mL of KM2 medium added with ciprofloxacin at the concentration of 1 MIC. The selection step was repeated as described above. At each selection step, the supplemented concentration of ciprofloxacin was increased twofold. The selection process was ended when the culture medium color showed no change (red to yellow) after three independent attempts. The MICs of parental *M. hyorhinis* and its developed FQs-resistant mutants selected at each step were determined using the broth microdilution method.

### 4.4. Analysis of Quinolone Resistance Determining Region

In order to analyze the mutations that occurred in the QRDRs, genomic DNAs of in vitro selected fluoroquinolone-resistant mutants of *M. hyorhinis,* and also the field isolates, were extracted from 20 mL logarithmic-phase broth cultures by using the Bacterial DNA kit (Omega). Gene fragments of QRDRs were amplified using specific primers (Table 3), which were designed according to the genomes of two *M. hyorhinis* strains: BTS-7 (NZ_KB911485.1) and HUB-1 (NC_014448.1). The PCR components of DNA templates (3 μL), pair of primers (2 μL, 10 μM), 2 × Phanta^®^ Max Master Mix (Dye Plus) (Vazyme, Nanjing, China) (25 μL), and ddH_2_O (18 μL) were mixed to a total volume of 50 μL system. The PCR reactions were performed with an i-Cycler (Biorad, Hercules, CA, USA) thermal cycler. The PCR conditions were conducted as follows. After 3 min at 95 °C, amplification was performed over 32 cycles, with 15 s at 95 °C, 15 s at 56 °C, and 1 min at 72 °C, with a final extension step of 5 min at 72 °C. PCR products were subjected to electrophoresis in 1% agarose gels containing 0.01% of TS-GelRed (Tsingke, Beijing, China). DNA bands were visualized under UV light. Subsequently, PCR products were purified using FastPure Gel DNA Extraction Mini Kit (Vazyme, Nanjing, China) and sequenced using an ABI 3730XI sequencer (ABI, Foster City, CA, USA). For convenience, the amino acid numbering refers to the *Escherichia coli* numbering and is based on the *E. coli* K12 sequences for GyrA (AAC7529.1), GyrB (AAT48201.1), ParC (AAC76055.1), and ParE (AAA69198.1).

## 5. Conclusions

Consistent with other reports, 25 strains of field *M. hyorhinis* isolates collected in Jiangsu, China, were most sensitive to tiamulin and tylvalosin. The in vitro developing process of fluoroquinolone resistance in *M. hyorhinis* was illustrated using stepwise increased concentrations of ciprofloxacin. The underlying molecular mechanisms included diverse and variable point mutations in the hot spots of GyrA, ParC, and ParE. The ParC subunit of topoisomerase IV might be the primary target of fluoroquinolones in *M. hyorhinis*. Revealing point mutations in the QRDRs could be useful for rapid detection of fluoroquinolone resistance in *M. hyorhinis* field isolates.

## Figures and Tables

**Table 1:** The background information and MIC data of the seven ciprofloxacin-sensitive *M. hyorhinis* isolates used for in vitro selection.

Strain	Origin	Year	MIC (μg/mL)								
			CIP	TIA	TYL	DOX	TVN	LIN	OTC	TIL	FFC
Mhr-JS-2010-36	Nanjing, China	2010	0.5	0.12	0.5	1	0.06	0.5	2	2	2
Mhr-JS-2010-37	Nanjing, China	2010	0.5	0.12	0.5	1	0.12	0.5	4	2	2
Mhr-JS-2011-40	Nanjing, China	2011	0.5	0.015	0.25	0.25	0.03	0.12	0.5	0.5	0.5
Mhr-JS-2014-43	Lishui, China	2014	0.5	0.25	1	0.12	0.12	1	0.25	4	1
Mhr-JS-2015-44	Nanjing, China	2015	0.5	0.12	0.5	0.5	0.12	0.5	2	2	2
Mhr-JS-2016-47	Liuhe, China	2016	0.5	0.25	0.5	1	0.06	1	2	4	8
Mhr-JS-2018-53	Taixing, China	2018	0.5	0.06	1	1	0.25	0.5	2	8	2

Note: CIP, ciprofloxacin; TIA, tiamulin; TYL, tylosin; DOX, doxycycline; TVN, tylvalosin; LIN, lincomycin; OTC, oxytetracycline; TIL, tilmicosin; FFC, florfenicol.

**Table 2:** The point mutations in the QRDRs of fluoroquinolones-resistant mutants collected at each selection step.

Stains	Initial MIC	QRDRs	Concentration of Ciprofloxacin for Selection (μg/mL)					
			0.5	1	2	4	8	16
Mhr-JS-2016-47	0.5	ParC	Ser80Phe	Ser80Phe	Ser80Phe	Ser80Phe	Ser80Phe	Ser80Phe
		ParE		Glu470Lys	Glu470Lys	Glu470Lys	Glu470Lys	Glu470Lys
		GyrA				Asp87Tyr	Asp87Tyr	Asp87Tyr
Mhr-JS-2018-53	0.5	ParC		Ser80Tyr	Ser80Tyr	Ser80Tyr	Ser80Tyr	
		ParE			Glu470Lys	Glu470Lys	Glu470Lys	
		GyrA					Ser84Pro	
Mhr-JS-2014-43	0.5	ParC		Ser80Tyr	Ser80Tyr	Ser80Tyr	Ser80Tyr	Ser80Tyr
		GyrA				Asp87Asn	Asp87Asn	Asp87Asn
Mhr-JS-2015-44	0.5	ParC			Phe80Tyr	Phe80Tyr	Phe80Tyr	Phe80Tyr
		GyrA				Ala83Val	Ala83Val	Ala83Val
Mhr-JS-2010-36	0.5	ParC		Glu84Gly	Glu84Gly	Glu84Gly	Glu84Gly	
		GyrA					Ala83Val	
Mhr-JS-2010-37	0.5	ParC		Glu84Lys	Glu84Lys	Glu84Lys	Glu84Lys	Glu84Lys
		GyrA			Asp87Asn	Asp87Asn	Asp87Asn	Asp87Asn
Mhr-JS-2011-40	0.5	ParC		Glu84Lys	Glu84Lys	Glu84Lys	Glu84Lys	Glu84Lys
		GyrA			Ala83Val	Ala83Val	Ala83Val	Ala83Val

**Table 3:** Primers and sequences of the genes coding for QRDRs.

Primer Target	Primer Sequence	Product Size (bp)
*gyrA*-F	ACTTCTTTTAAATTATGAGGG	621
*gyrA*-R	TGAAGCAGAACTAGAACAA	
*gyrB*-F	CACAGATAGTTATTCTGATTC	831
*gyrB*-R	GGTTGAGCTATGTAAACAT	
*parC*-F	ATGAAGAAACTAGATAATAATATG	609
*parC*-R	TTCTATACAAGCATCAATTA	
*parE*-F	AATTAAACATTCAAATCCAATT	670
*parE*-R	TGAATATGCATAAACAACTT	
